# Who continues to work after retirement age?

**DOI:** 10.1186/s12889-024-18161-1

**Published:** 2024-03-04

**Authors:** Sarah Zaccagni, Anna Munk Sigsgaard, Karsten Vrangbaek, Laura Pirhonen Noermark

**Affiliations:** 1 Department of Prevention, Health Promotion and Community Care, Steno Diabetes Center Copenhagen, Herlev, Denmark; 2 Department of Economics & Business Economics, Aarhus University, Aarhus, Denmark; 3 Department of Public Health, University of Copenhagen, Copenhagen, Denmark

**Keywords:** Retirement, Extending working life, Survey data

## Abstract

**Background:**

Demographic changes in all industrialized countries have led to a keen interest in extending working lives for older workers. To achieve this goal, it is essential to understand the patterns of retirement and specifically what characterizes individuals who continue to work beyond retirement age. Thus, the aim of this paper was to contribute to the international body of empirical knowledge about individuals who continue in the workforce after retirement age. We present evidence from Denmark and examine what characterizes individuals who continue in the workforce after retirement age and investigate the likelihood of continued work after retirement age while controlling for a set of socio-economic and lifestyle factors.

**Methods:**

The study population consisted of 5,474 respondents to the Copenhagen Aging and Midlife Biobank (CAMB) 2021 survey, divided into two groups. The first group included subjects (*n* = 1,293) who stayed longer in the workforce even though they had the possibility to retire. The second group consisted of subjects who had retired full-time at the time of the survey (*n* = 4,181). Survey data was linked to register data to provide a broader dataset. In order to investigate the heterogeneity between the two groups in terms of important socio-economic, work-related and health-related variables, t-test, Mann-Whitney U (Wilcoxon Rank) test, and chi-square tests were employed. Further, to examine the probability of an individual working after retirement age a logit model with step-wise inclusion was utilized.

**Results:**

Overall, individuals who continue to work even though they could retire tend to be wealthier, healthier, and males compared to individuals who are retired full-time. Further, there are more older workers who have partners and are co-habitants than retirees. The likelihood of continuing in the workforce past retirement age is affected by several work-related factors as well as life-style factors. The likelihood of working past retirement age decreases by years spent in the workforce (marginal effect of -0.003), if you have a partner (-0.080) and if your partner is outside of the workforce (marginal effect of -0.106). The likelihood increases by health (marginal effect of -0.044 of moving from excellent/very good health to good health or to fair/poor health, physical working capability (marginal effect of -0.083 of moving from no/some problems to severe problems or cannot work at all) and income (marginal effect of 0.083 from moving from the lowest income-quantile to higher quantiles).

**Conclusion:**

These results are in line with the previous literature and suggest the importance of designing retirement policies that tailor the transition toward retirement according to specific characteristics of both the individual and the segment of occupation.

**Supplementary Information:**

The online version contains supplementary material available at 10.1186/s12889-024-18161-1.

## Introduction

An increase in the ageing population is an emerging issue in many parts of the world. The rise in the number of older persons will increase both the need for healthcare resources and the economic burden on society due to an increase in pension benefit payments. To mitigate some of the burden on society an extension in working lives is often proposed as a solution [1, 2]. Not only can the extension of working lives result in an increase in total hours worked and labor supply but also be beneficial in terms of increases in quality of life, mental health and health in general for older workers [3–16]. However the extension of working lives can also have the opposite effects, some studies have found prolonged working life to have no significant effects on health-related outcomes [17–19] or inconclusive effects [20] and some have even found negative effects [21–26]. Important to keep in mind is that not all older workers are willing or able to work past (or up until) retirement age which requires a well-designed and flexible retirement system. In order to design and evaluate retirement policies that takes individuality into account while extending the working life of older workers it is imperative to have information regarding individuals’ retirement timing and reasons for retirement and most importantly, baseline information about who continues in the workforce past retirement age in the current system.

Previous research on what characterizes individuals that extend working life beyond retirement age point to several important factors. A systematic literature review finds being female, having a burn-out and the presence of discrimination against older workers to be negatively associated with prolonged working life while higher education, being married, having intrinsic motivation and work flexibility to be positively associated with working after retirement age [27]. Other previous research also emphasize the important role of education. Education later in life has been shown to increase the propensity to stay in the workforce in a Swedish setting [28]. In a multi-country study, highly educated women were found to have higher odds of employment at older ages compared to women with a lower education [29]. In a similar manner, in a study from Japan, men who had a higher education were more likely to choose to prolong working life compared to their less educated counterparts [30]. However, occupational training has not been shown to affect retirement timing [31].

Economic incentives are an essential aspect for older workers in the decision-making process surrounding retirement timing [32]. A Dutch study on older persons in paid employment showed that individuals in a poor financial situation worked beyond retirement age more often than those in a better economic situation [32]. Therefore, income from work, wealth and pension benefits are factors that need to be considered when investigating who retires and who continues in the workforce after retirement age. Similarly, individuals who continue in the workforce due to enjoyment of work have been shown to have a higher level of quality of life compared to individuals who continue work in older ages due to financial constraints [22]. Hence, financial aspects, are essential to include in analyses surrounding retirement and retirement pathways.

The health of an individual often decides if a person is going to work beyond retirement age, retire early or retire at retirement age [30, 32, 33]. Persons in good health, both physically and/or mentally, have a higher likelihood of participating in the workforce after retirement age. A study conducted in Finland which used both survey and register data found good mental health to be an important determinant behind the extension of working lives for individuals close to retirement age [34]. Further, also employing register data, a study from the US indicated that men over the age of 70 had a higher probability of staying in the workforce after retirement age if they were in excellent health compared to poor health [35]. Differences have also been found in the predictors of working beyond retirement between workers with chronic diseases and workers without chronic diseases [32]. In a slightly different manner, health has been shown to play an important role in the choice of whether to engage in bridge employment [36, 37]. A scoping review on the incentives for prolonged working life summarizes that individuals in good health, with high education and interest in monetary gain from employment are more likely to extend their working life beyond retirement age compared to their counterparts [38].

Other studies point to additional factors for prolonged working life. Gender seems to play an important role for the patterns of continued work after retirement age [39–41]. A descriptive study including four different countries concluded that men are more likely to continue in the workforce after retirement compared to women [42]. Further, a partner’s or a close friend’s retirement age and attitude towards retirement seems to matter when individuals decide on when to retire [43]. Thus, including variables describing civil status, and if possible, the occupational status of a partner in analyses will provide important information when examining retirement patterns for older workers. Older workers who enjoy and appreciate their work and are highly engaged or interested in their work are the individuals who more often work past retirement age [32]. Further, older workers who felt that their job positively affected other people had more reasons to stay in employment after retirement age [44]. Similarly, good working conditions have been shown to be a prerequisite for older workers to continue working after the age of 65 [45].

While these studies provide a growing body of knowledge about retirement patterns and dynamics, they are still limited in terms of their national contexts and polity scope.– It is therefore important to extend the number of empirical studies from different contexts to solidify our understanding of retirement dynamics.

The Nordic region, including Denmark, represents a set of relatively prosperous countries where the extensive welfare services and the combination of state and labor market pensions means that economic constraints are unlikely to be the dominant reason for continuing to work beyond retirement. This means that we can obtain a clearer picture of the basic motivational factors for continued work from this region. In this study we utilize detailed survey and registry data from Denmark to investigate a broad range of theoretically relevant factors. We argue that results from the Nordic region, including Denmark, about motivational factors are likely to also be relevant in many other countries in situations where continued work is not primarily motivated by economic necessity for most citizens. The Danish case is particularly interesting in regard to gender issues. Previous studies have shown that males are much more likely to continue formal work after retirement. This has been explained by gender differences in employment history and job types, and by higher female responsibilities for informal care giving. It is relevant to investigate whether such results are supported in the Danish case where female workforce participation is high, and where the extensive welfare services and universal health care should reduce the need for informal caregiving.

A deeper and broader understanding of retirement patterns and motivations is crucial to optimize policy efforts, to save resources and to stimulate workplaces that can support the willingness to continue working in older ages. Therefore, the aim of this paper is to contribute with empirical knowledge about what characterizes individuals who continue in the workforce after retirement age. We present novel empirical evidence from Denmark, which is one of the Nordic welfare states characterized by universal health coverage, publicly funded long-term care and a relatively long tradition for combining public pensions, labor market pensions and private pensions in a flexible pension system.

## Institutional background

The Danish pension system is designed in a manner that makes it possible to receive pension from multiple sources. Overall, the system can be divided into three different pillars where pillar one contains the statutory pensions, which are pensions that are financed through taxes and controlled by the public sector. Statutory pensions primarily include the public old age pension (*folkepension*), which is a universal residence-based benefit scheme that secures a minimum pension for persons who reach the state retirement age. Entitlement and level of pension depends on citizenship (or refugee status). You must have lived in Denmark for at least 40 years to obtain full pension. Persons that live alone get higher public pensions and there are various conditions that may entitle you to supplementary public pensions (e.g. high out of pocket health expenditures or heating expenditures above a given threshold). Disability pensions are also included in the first pillar, and entitled for citizens whose capacity is permanently reduced to such a degree that they are unable to work (often limited to people above the age of 40 years). The amount you receive in disability pension depends on different factors such as marital status and other household income. The state pension age depends on which year you were born and has gradually been raised through the years. For people born 1953 or earlier the public retirement age is 65 years and for people born 1967 or later the public retirement age is 69 years (depending on future indexations). For approx. 50% of Danish pensioners, pillar one coverage is the only source of income during retirement.

The second pillar contains the privately funded labour market pension schemes, which are contribution-defined and set up as part of your employment contract. Almost all Danes have this type of pension saving. Tax-financed earnings-related civil servant pensions are also included in this pillar but are planned to be phased out.

The third pillar consists of individual pension schemes. These are voluntary, tax-deductible individual pension savings that go beyond the occupational pension schemes. In addition to the three pension pillars there are two possibilities for publicly supported voluntary early retirement pension (VERP). The *first* provides access to early retirement payments for up to three years before reaching the age for obtaining public pensions. This scheme is restricted to persons that have contributed to a special early retirement scheme for at least 30 years and are members of an unemployment insurance fund and are active in the labor market. The payment is limited to the maximum level of unemployment benefits and there is a deduction for other labor market and private pensions. These restrictions were introduced in a pension reform in 2011 aiming to increase the labor supply and to target persons with limited pension savings. The *second* scheme was introduced in 2011. It provides the right to early retirement for up to three years before the age of retirement if you have been active in the labor market for at least 40 years. The payments in this scheme are lower, and it is specifically targeted towards persons that have entered the labor market early and have not contributed to the special early retirement scheme. Finally, if you are assessed as fully or partially unable to work, you can be granted a specific early pension for up to six years before being entitled to public pension.

The early retirement pension schemes have been reformed in 2011 and 2023 in response to increasing life expectancies and lower birth rates which challenge the demography in the labor market.^1^

## Methods

### Material

This study utilizes both survey- and register data. Survey data originates from the Copenhagen Aging and Midlife Biobank (CAMB) follow-up study. CAMB is a Danish population-based Ageing Cohort study based on three pre-established cohorts, two of which have been followed since their birth in 1953 (the Metropolit Cohort, males born in Copenhagen) and in 1959-61 (the Copenhagen Perinatal Cohort, men and woman born in Copenhagen). The third cohort includes people born in 1948-49 and 1958-59 (the Danish Longitudinal Study on Work, Unemployment, and Health (DALWUH) cohort, a random sample of the Danish population in 1999). All participants in the pre-existing cohorts, living in the Eastern part of Denmark, were invited to the CAMB study in 2009–2011 (*N* = 17,937) to establish a database to contribute with knowledge on the life course determinants of age-associated changes in health in mid- and late life [46, 47]. In the spring of 2021, all original cohort members (aged 59–73 years) were invited to the CAMB follow-up survey (*N* = 24,133) with a special focus on retirement. The data collection took place from 12. April 2021 to 13. June 2021 administered as a web-based survey sent out to eligible participants (alive and living in Denmark, without address protection and with a permanent address). In total, 10,275 (42.58%) responded to the survey.

All participants were linked to the national registers at Statistics Denmark through a unique Civil Registration Number (CPR). Register data from Statistics Denmark was employed in order to verify self-reported survey data and to supplement with information that was not included in the survey data. The registers utilized were the register for education (Uddanelsesregistret UDDA), the population register (Befolkningsregistret BEF) and the income register (Indkomstregistret IND).

### Study population

**Fig. 1:**
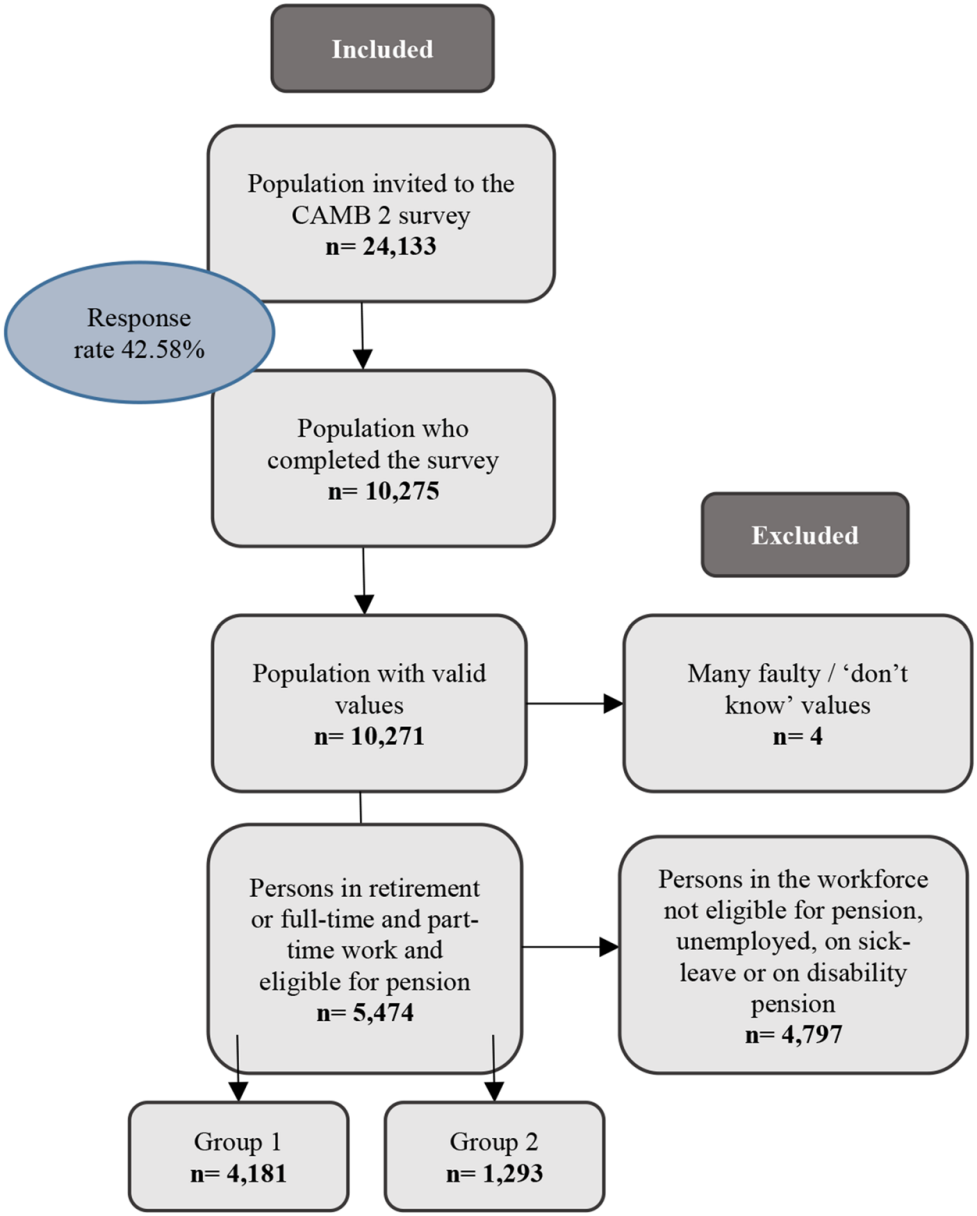
Sample definition of study participants

The study population consists of 5,474 individuals from the CAMB survey 2021 (wave 2), individuals who are retired full-time at the time of the survey and older workers. Individuals retired full-time was chosen as the comparable to be able to have a clear control group to compare against the older workers. See Fig. 1 below for an overview of the sample definition of the study population. The first group includes individuals who have answered “Retired full-time” to the question “What is your labor market affiliation?” (*n* = 4,181). The second group includes individuals who have chosen to stay longer in the workforce even though they had the possibility to retire. This group was based on participants who answered that they are either a “full-time employee (more than 30 hours per week)”, “part-time employee (less than 30 hours a week) and do not receive pension”, “self-employed (have my own company)” or in a “flex job” and answered “yes” to the question “Can you choose to retire, but have chosen to continue working?” (*n* = 1,293).^2^

### Measures

In order to describe the two different groups using descriptive statistics, data from both the CAMB survey and the national registers were employed. From the CAMB survey, information on demographics, health and wellbeing, memory, employment, retirement, civil status, and health behaviors were selected.

More specifically, the included variables were: (1) from the demographic section: age, civil status and cohabitation; (2) from the health and wellbeing section: health, stress, quality of life, physical working capability, falls, height and weight, and memory loss; (3) from the labor market section: labor market affiliation and the partner’s labor market affiliation; (4) from the employment section: years at current/most previous workplace, subordinates, engagement in unpaid work and night work; (5) from the social network section: children, (6) from the health behaviors section: physical activity, sleep, smoking and alcohol drinking.

From the national registers the following variables were included: (1) from the education register, UDDA: highest educational attainment level; (2) from the population register, BEF: gender and region; (3) and from the income register, IND: wealth measured as weighted household income. All employed variables from the registers were measured for the year 2020 which was the most recent data during the time of data collection.

### Statistical analysis

We explore the heterogeneity of the characteristics across the two populations that we described above using a set of parametric and non-parametric tests, based on the variable type (i.e., whether the variables are categorical, ordinal or interval and whether they are normally distributed). Specifically, the tests that we employed were: (1) Test of proportion for binary variables, (2) T-test for continuous variables, (3) Chi-square test for categorical variables, and (4) Mann-Whitney U-test for ordinal variables. For continuous variables, checks for normal distribution were conducted using a Shapiro Wilk W-test, and when the normality requirement was not satisfied the results were checked employing the parametric t-test using Mann-Whitney U-test. A correlation matrix of all the variables can be found in Table A1 in the Appendix.

#### Logistic regression

A logistic regression model was chosen as the statistical analysis model. In our case, the model provides probabilities that an individual participates in the workforce after being able to retire while controlling for a set of independent variables. Logistic regression was employed using the binary variable if an individual was participating in the workforce (fully or partly) after having the possibility to retire or not (having a value of 0 if the individual was retired and a value of 1 if the individual continued in the workforce even though he or she could be in retirement) as the dependent variable. In order to decide on which characteristics that are important to analyze in answering the aim of this study, previous published literature was used when choosing the independent variables for the regression model. As previously stated, research surrounding retirement timing has shown that individual characteristics important in this area of research are age, health, civil status, gender, education, and economic incentives. Therefore, the independent variables that were chosen to explain the relationship between individual characteristics and retirement timing were gender, age, education level, income level, civil status, self-reported health (both in general and at work), employment conditions, health behaviors and physical activity. The variables that were found not to meet the conditions of heterogeneity were excluded from the regression model. The logistic regression then reported the marginal effects (using the “mfx” command in STATA) of continuing in the workforce beyond retirement age while controlling for individual socio-economic, life-style, health-related characteristics. To see how statistical significance and the magnitude in coefficients and marginal effects differ when controlling for different types of variables, step-wise inclusion in the model was implemented.

## Results

Below, the results from the statistical test of difference in means are presented in detail (for a full overview, see Table 1). Thereafter, the results from the logistics regression are accounted for.

**Table 1:** Descriptive statistics of retirees (group 1), older workers in the workforce after retirement (group 2)

Variable name (Characteristics)	Group 1 *n* = 4181 (76.38)	Group 2 *n* = 1293 (23.62)	Total *N* = 5474 (100)	Source	Test	p-value
**Gender, n (%)**				Register	X^2	< 0.0001
Man	3189 (76.27)	1089 (84.22)	4278 (78.15)			
Woman	992 (23.73)	204 (15.77)	1196 (21.85)			
Missing	0	0	0			
**Age, Mean (SD)**	69.44 (2.51)	66.39 (3.49)	68.72 (3.06)	CAMB	T-test/ MW-test	< 0.0001/ < 0.0001
Missing	7	< 5	< 12			
**Health, n (%)**				CAMB	X^2	< 0.0001
Excellent/very good	1734 (41.47)	753 (58.24)	2487 (45.43)			
Good	1760 (42.10)	446 (34.49)	2206 (40.30)			
Fair/poor	687 (16.43)	94 (7.27)	781 (14.27)			
Missing	0	0	0			
**Partner, n (%)**				CAMB	X^2	< 0.0001
Partner	3376 (80.75)	1125 (87.00)	4501 (82.22)			
No partner	805 (19.25)	168 (13.00)	973 (17.78)			
Missing	0	0	0			
**Living situation, n (%)**				CAMB	X^2	< 0.0001
With someone	3167 (75.75)	1061 (82.06)	4228 (77.24)			
Alone	1014 (24.25)	232 (17.94)	1246 (22.76)			
Missing	0	0	0			
**BMI, n (%)**				CAMB	X^2	< 0.0001
< 18.5	27 (0.65)	11 (0.86)	38 (0.70)			
18.5-<25	1488 (35.95)	451 (35.21)	1939 (35.76)			
25-<30	1766 (42.67)	610 (47.62)	2376 (43.86)			
30+	858 (20.73)	209 (16.32)	1067 (19.68)			
Missing	42	12	54			
**Smoking, n (%)**				CAMB	X^2	0.258
Smokers	571 (14.02)	158 (12.75)	729 (13.72)			
Non-smokers	3503 (85.98)	1081 (87.25)	4584 (86.28)			
Missing	107	54	161			
**Physical activity, n (%)**				CAMB	X^2	< 0.0001
< 3 h/weekly	464 (11.39)	187 (15.09)	651 (12.26)			
3 + hours/weekly	3608 (88.61)	1052 (84.91)	4660 (87.74)			
Missing	109	54	163			
**Educational level, n (%)**				Register	X^2	< 0.0001
Low	614 (14.78)	137 (10.64)	751 (13.80)			
Medium	1958 (47.12)	531 (41.23)	2489 (45.73)			
High	1583 (38.10)	620 (48.14)	2203 (40.47)			
Missing	26	5	31			
**Region, n (%)**				Register	X^2	< 0.0001
North Jutland	249 (5.96)	50 (3.87)	299 (5.46)			
Central Jutland	561 (13.42)	140 (10.83)	701 (12.81)			
Southern Denmark	570 (13.63)	131 (10.13)	701 (12.81)			
Copenhagen	1907 (45.61)	710 (54.91)	2617 (47.81)			
Zealand	894 (21.38)	262 (20.27)	1156 (21.12)			
Missing	0	0	0			
**Fallen within the last couple of months, n (%)**				CAMB	X^2	< 0.0001
Yes	806 (19.28)	182 (14.08)	988 (18.05)			
No	3375 (80.72)	1111 (85.92)	4486 (81.95)			
Missing	0	0	0			
**Limited by your health (work related), n (%)**				CAMB	X^2	< 0.0001
No problems/some	3830 (91.60)	1261 (97.53)	5091 (93.00)			
Severe difficulties/cannot work at all	351 (8.40)	32 (2.47)	383 (7.00)			
Missing	0	0	0			
**Quality of life, n (%)**				CAMB	X^2	< 0.0001
Very good/good	3132 (74.91)	1118 (86.47)	4250 (77.64)			
Neither good/bad	713 (17.05)	135 (10.44)	848 (15.49)			
Fair/poor	336 (8.04)	40 (3.09)	376 (6.87)			
Missing	0	0	0			
**Stressed, n (%)**				CAMB	X^2	< 0.0001
All the time/much of the time	117 (2.80)	50 (3.87)	167 (3.05)			
A part of the time	279 (6.67)	129 (9.98)	408 (7.45)			
A little of the time	1365 (32.65)	519 (40.14)	1884 (34.42)			
At no time	2420 (57.88)	595 (46.02)	3015 (55.08)			
Missing	0	0	0			
**Memory loss, n (%)**				CAMB	X^2	< 0.0001
To a great extent	91 (2.18)	16 (1.24)	107 (1.95)			
To some extent	1785 (41.69)	409 (31.63)	2194 (40.08)			
No	2305 (55.13)	866 (66.98)	3173 (57.96)			
Missing	0	0	0			
**Partner’s labor market affiliation, n (%)**				CAMB	X^2	< 0.0001
Employed	641 (19.05)	681 (60.59)	1322 (29.45)			
Unemployed	41 (1.22)	32 (2.85)	73 (1.63)			
Sick leave from work	22 (0.65)	12 (1.07)	34 (0.76)			
Retired w. part time work	141 (4.19)	63 (5.60)	204 (4.54)			
Retired incl. VERP	2520 (74.89)	336 (29.89)	2856 (63.52)			
Missing	816	169	985			
**Do/did you have any subordinates?, n (%)**				CAMB	X^2	< 0.0001
Yes	1496 (36.01)	375 (29.14)	1871 (34.39)			
No	2658 (63.99)	912 (70.86)	3570 (65.61)			
Missing	27	6	33			
**Do you have children?, n (%)**				CAMB	X^2	0.044
Yes	3434 (83.21)	1087 (85.59)	4521 (83.77)			
No	693 (16.79)	183 (14.41)	876 (16.23)			
Missing	54	23	77			
**Have you ever had night work at least 3 nights a month?, n (%)**				CAMB	X^2	0.696
Yes	1440 (34.97)	451 (35.57)	1891 (35.11)			
No	2678 (65.03)	817 (64.43)	3495 (64.89)			
Missing	63	25	88			
**Household income, mean (SD)**	315173.3 (445805.2)	524398.4 (863941)	364456.8 (579235.6)	Register	T-test/ MW-test	< 0.0001/ < 0.0001
Missing	< 5	5	< 10			
**Years in latest employment, mean (SD)**	29.28 (14.10)	19.96 (14.57)	27.07 (14.76)	CAMB	T-test/ MW-test	< 0.0001/ < 0.0001
Missing	71	16	87			
**Hours spent on voluntary work, mean (SD)**	2.28 (5.22)	1.82 (5.02)	2.17 (5.18)	CAMB	T-test/ MW-test	0.0068/ 0.0028
Missing	59	25	84			
**Weekly alcohol consumption, mean (SD)**	12.68 (14.64)	11.89 (10.82)	12.49 (13.84)	CAMB	T-test/ MW-test	0.0705/ 0.6552
Missing	203	76	279			

BMI = Body Mass Index; CAMB = Copenhagen Aging and Midlife Biobank; MW = Mann-Whitney; SD = Standard deviation; VERP = Voluntary early retirement pension

### Socio-economic & demographic information

**Gender.** The population is composed by 78.15% of men (*n* = 4,278); the gender imbalance is due to the fact that a cluster of the CAMB population belongs to the male-only cohort (Metropolit cohort). Looking at gender, men tend to continue to work after they reach the threshold for retirement significantly more than women. Out of 4,278 men, 1,089 work after retirement and 3,189 do not. Out of 1,196 women, only 204 work after retirement and 992 do not (82.9%).

**Age.** The age of the group who continues to work is significantly lower (66.39) than the average age in the group of those who are retired (69.44).

**Partner and Co-living.** Having a partner seems to play a positive role in determining the decision to continue to work. Among those who declare to have a partner 25% continue to work and 75% do not; while among those who do not have a partner, the percentage of those who continue to work is significantly lower (17.3%). Similar results are found when looking at subjects living with someone as compared to living alone.

**Region.** For the geographical distribution of subjects in the sample, the older workers live in the metropolitan area of Copenhagen to a greater extent than the retirees.

**Educational level.** Individuals who are in the work force after being able to retire have a higher education compared to individuals who have retired. There are also fewer older workers in the group with the lowest educational level compared to retirees.

**Household income**. Individuals who continue in the work force have a significantly higher household income (proxy for wealth) compared to individuals who are retired.

### Working-conditions and environment

**Partner’s labor market affiliation.** Over 60% of the older workers have partners that are in the work force while only 19% of the retirees have partners that are employed. In the same manner, 75% of retirees reported that their partners are also retired and only 30% of the older workers have partners in retirement.

**Subordinates.** More individuals in the retired group reported that they had subordinates when they were in the work force than individuals in the older worker group.

**Years in latest employment.** Individuals who are retired had been employed 10 years longer (on average 29 years) at their most recent workplace compared to older individuals in the work force who could have retired but are still in the work force.

**Ability to work limited by health.** More individuals that are in the work force report that their health causes no problems or some problems in relation to the work that they do compared to those individuals that are retired.

### Health and lifestyle

**Health.** The percentage of individuals who reported to be in excellent health or very good health is significantly higher (58%) among those who continue to work after retirement compared to those who are retired (41%).

**Body Mass Index (BMI).** Among individuals who are retired a significantly higher percentage are obese (BMI > 30) compared to the population of older workers.

**Physical activity.** The percentage of individuals who exercise 3 h or more per week is higher among those who are retired compared to the older workers.

**Quality of life.** A larger share of the older individuals still in the work force report to have very good or good quality of life compared to their retired counterparts while more individuals from the latter group report to have neither good nor bad or fair/poor quality of life.

**Memory loss.** More individuals in the retired group have answered that they feel that their memory has declined substantially “to a great extent” or “to some extent” compared to the individuals still in the work force. Subjects who continue to work are more likely to answer that they have not experienced a decline in their memory compared to those who retire (67% vs. 55%).

**Falls.** Individuals who reported that they fell during the past months are more numerous in the retired group compared to the work force group.

**Stress.** Older workers reported to a greater extent that they were feeling stressed compared to their retired counterparts. The number of those who declare to be stressed “at no time” is higher in the retired group.

#### Logistic regression

The probability of an individual continuing in the workforce past retirement (the dependent variable) was investigated using a Logit model following a step-wise inclusion of independent variables. First, the probability of participation in the workforce dependent on individual health was estimated, thereafter age and gender were included. In the third model income and education were included, in the fourth specification variables on individual network, in the fifth health-related and life-style variables, and in the last model work-related variables were included. The results are reported in Table 2 below. Here, both the coefficients from the Logit model as well as the marginal effects are reported. In short, increasing age decreases the probability that an individual is in the workforce after retirement– a 1 year increase in age decreases the probability of the individual continuing in the workforce by between 0.054 and 0.031. The more an individual consumes alcohol in one week the smaller the probability that the individual is an older worker and the same is true for physical activity (marginal effects − 0.001 and 0.083 respectively). Also, the longer the individual has participated in the workforce the lower is the probability that he or she is still in the workforce after retirement with a decrease in probability of 0.003 per year worked. This is also the case for the group of women compared to the group of men, a decrease in probability of participation in the workforce after retirement age of 0.043 for a woman compared to a man. The probability of working after retirement increases with higher wealth (marginal effect 0.083) but decreases with higher education and if the person has subordinates (marginal effect − 0.018 and 0.068 respectively). Lastly, the healthier an individual is the higher the probability that the individual is still in the workforce, both in terms of general health (marginal effect − 0.044 from moving from excellent/very good health to fair/poor health) but also physical working capability (the extent to which health limits your work) with a marginal effect of -0.083 from moving from no problems/some problems working to severe problems/cannot work at all.

**Table 2:** Results from logistic regression with several models included (step-wise inclusion of independent variables) using continued work after retirement as the dependent variable (0 if not continued in the workforce past retirement, 1 if continued in the workforce past retirement)

	Model 1 (obs. 5474) Only health		Model 2 (obs. 5465) With age and gender		Model 3 (obs. 5428) With income and education		Model 4 (obs. 5353) With partner and children		Model 5 (obs. 5109) With life-style and health-related factors		Model 6 (obs. 5012) With all explanatory variables	
	Coefficient	Marginal effects	Coefficient	Marginal effect	Coefficient	Marginal effect	Coefficient	Marginal effect	Coefficient	Marginal effect	Coefficient	Marginal effect
**Health** (Reference: excellent/very good health; coded: 1–3)	-0.562*** (0.049)	-0.099*** (0.009)	-0.581*** (0.054)	-0.087*** (0.008)	-0.330*** (0.059)	-0.042*** (0.007)	-0.359*** (0.060)	-0.045*** (0.008)	-0.449*** (0.065)	-0.055*** (0.008)	-0.392*** (0.072)	-0.044*** (0.008)
**Age**			-0.362*** (0.014)	-0.054*** (0.002)	-0.328*** (0.015)	-0.041*** (0.002)	-0.329*** (0.015)	-0.041*** (0.002)	-0.321*** (0.016)	-0.039*** (0.002)	-0.279*** (0.017)	-0.031*** (0.002)
**Gender** (Reference: male; coded: 0–1)			-0.584*** (0.114)	-0.078*** (0.013)	-0.459*** (0.123)	-0.053*** (0.013)	-0.472*** (0.124)	-0.054*** (0.012)	-0.495*** (0.130)	-0.055*** (0.013)	-0.417*** (0.140)	-0.043*** (0.013)
**Household income (wealth)** (Income in quantiles)					0.743*** (0.034)	0.094*** (0.004)	0.779*** (0.036)	0.097*** (0.004)	0.803*** (0.037)	0.098*** (0.004)	0.735*** (0.040)	0.083*** (0.004)
**Education** (Reference: low 1 education; coded: 1–3)					-0.185*** (0.060)	-0.023*** (0.008)	-0.188** (0.061)	-0.023** (0.008)	-0.143** (0.064)	-0.017** (0.008)	-0.161*** (0.067)	-0.018*** (0.008)
**Children** (Reference: yes 1; coded: 1–2)							-0.058 (0.111)	-0.007 (0.014)	-0.094 (0.116)	-0.011 (0.014)	-0.064 (0.121)	-0.007 (0.014)
**Partner** (Reference: No (0); coded: 0–1)							-0.284 (0.204)	-0.038 (0.029)	-0.299 (0.213)	-0.039 (0.030)	-0.610*** (0.226)	-0.080** (0.034)
**Living situation** (Reference: No (0); coded: 0–1)									-0.163 (0.187)	-0.021 (0.024)	-0.115 (0.197)	-0.013 (0.023)
**Alcohol consumption** (Weekly consumption in number of units)									-0.010*** (0.004)	-0.001*** (0.000)	-0.009** (0.004)	-0.001** (0.000)
**Falls** (Reference: Yes 1; coded: 1–2)									0.092 (0.113)	0.011 (0.014)	0.090 (0.120)	0.010 (0.014)
**Physical activity** (Reference: less than 3 h weekly 1; coded: 1–2)									-0.698*** (0.124)	-0.085*** (0.015)	-0.734*** (0.132)	-0.083*** (0.015)
**Smoking** (Reference yes 1; coded: 1–2)									-0.118 (0.123)	-0.014 (0.015)	-0.032 (0.130)	-0.004 (0.015)
**BMI**									0.082 (0.059)	0.010 (0.007)	0.083 (0.062)	0.009 (0.007)
**Voluntary work** (Hours weekly)											-0.013 (0.008)	-0.001 (0.001)
**Years worked**											-0.028*** (0.003)	-0.003*** (0.000)
**Subordinates** (Reference: yes 1; coded: 1–2)											0.599*** (0.094)	0.068*** (0.011)
**Physical working capability** (Reference: no/some problems (0); coded: 0–1)											-1.012*** (0.251)	-0.083*** (0.014)
**Partner’s labor market status** (Reference: In workforce; coded: 0–1)											-0.805*** (0.097)	-0.106*** (0.015)

Standard errors in parentheses; *** *p* < 0.01, ** *p* < 0.05, * *p* < 0.1

## Discussion

Changing demographics in populations, in this instance an increase in the older part of the population due to increased life expectancy, creates the need for an expansion of the labor force in order to tackle increases in the number of paid pension benefits. One solution widely suggested is the extension of working lives of older workers. To be able to account for individual differences in the ability and willingness to work past retirement age and, as a result design fair and optimal retirement policies, research surrounding who continues to work past retirement age is needed.

In this study, we compare a group of individuals that have retired full-time with individuals that are still in the workforce even though they could have retired. For our comparison, we use a set of socio-economic, lifestyle, work-related and health-related factors. Further, we calculate the probability of an individual being in the workforce after retirement while controlling for factors demonstrated to be important from previous literature in the field. To facilitate this, we employ survey- and register data from Danish older workers and retirees. We find that older workers experience less falls and less memory loss, are in better health and have better quality of life compared to retirees. Older workers also have partners and are co-habitants more frequently than individuals that have retired. Further, the probability that an individual is still in the workforce after retirement increases with wealth, health, the presence of subordinates at work and if the individual is a male compared to a female. The same probability decreases with age, years spent in the work force, education level, alcohol consumption and physical activity.

The health of an individual seems to be one of the most important factors determining whether an individual is in the work force after retirement age or has retired. More specifically, and to no surprise, the individuals that are in better health are more frequently participating in the work force after retirement age compared to individuals in worse health [32, 34–37]. Our findings corroborate what has been found in previous studies. We find that individuals that are in the work force after retirement age more often report being in excellent or good health compared to individuals that are retired full-time. In addition, our results show that the probability of being in the work force after retirement age increases with better health. It is often unclear whether individuals in good health are the ones continuing work after retirement or if work after retirement leads to good health [23]. If a positive association of continued work on health is observed it might be the case that unobserved or uncontrolled variables influence health, not continued work per se. For example, if highly educated individuals have a higher probability of choosing continued work and individuals with a higher education have better health compared to their counterparts the positive association of continued work on health would, at least partly, be caused by other factors than just continuing in the workforce. Further, highly educated individuals could have a more positive approach when reporting self-assessed health compared to individuals with less education [23]. In addition, stating the obvious, it is highly likely that individuals in better health continue in the workforce compared to those in poorer health. This phenomenon is typically called the “healthy worker effect” and when this problem is not accounted for results are likely to be biased [48]. We include other health-related variables with the aim to investigate the relationship between health and continued work in more depth. We find that those who are retired full-time have a higher BMI and have more falls compared to older workers. In addition, better work-related health (how health affects an individual’s ability to work) increases the probability of staying in the workforce. After including other health- and life-style related factors, we find that the probability of being in the workforce decrease with increased physical activity. This might be because retirees have more free time compared to those who work (since we are looking at data related to the same year).

Another factor found to influence continued workforce participation is gender. A large part of the literature surrounding retirement transitions concludes that men are more often working after retirement age compared to women [39, 42]. We also found this to be the case for our Danish study population. Our results showed that males are overly represented in the work force compared to women. Further, the probability that an individual works after being able to retire increases if the older worker is a male compared to being a female. The cause of this could be that men are perhaps more often employed in industries where it is possible and encouraged to continue working past retirement age. Moreover, because most of the informal caregiving in society is provided by women it could, and most likely does, affect women’s decision-making in retirement transitions [49–51].

The level of an individual’s education affects the presence in the work force after retirement age and research shows that the higher the education, the more probable it is that the individual is working after retirement age. In this study, individuals that continue in the workforce have a higher educational level compared to their retired counterparts. However, investigating this matter using our logit model shows that the probability of being in the workforce decreases with education level (the same case of opposite signs occurs for the variable “subordinates”). This should be interpreted with caution and most likely is a result of the assumptions underlying the logistic regression model.

The overall findings in this study point towards the differences in the older population in terms of who continues in the work force and who retires full-time. The individuals that continue in the work force are wealthier, healthier, and males. These differences need to be considered when designing retirement policies on a national level but also on an organizational level. On an individual level some of these factors are exogenous and some are difficult for an individual to influence however, gaining knowledge on what factors contribute to a long work life could affect individual decision making during the life course. At the overall policy level, it is important to acknowledge the differences in propensity to continue working. This means that policies should be sufficiently flexible to allow individual choice. Another general policy recommendation is that particular emphasis should be placed on providing support to families to reduce the burden of informal care giving, which has been found to fall disproportionately on women, even in the Nordic welfare states. A final policy recommendation is that it is important to support workplaces in providing physical and psychological work environments that can enable persons in less-than-optimal health to continue working. This could lower the threshold for continued work for persons that would otherwise leave the formal job market.

There are some strengths and limitations of the current study that are important to emphasize. We utilize data from a large dataset that includes both survey and register data. Having access to survey data provides a broader and more nuanced picture of retirement transitions and decisions as well as information on life-style factors that are not available in national registers. However, a large dataset and data sources do not fully solve the problem of causality and the healthy worker-effect. In our attempt to account for this issue we include all available and relevant observable individual factors but of course there are unobserved factors that we cannot account for. In addition, we could not observe characteristics of the individuals that have not responded to the survey and therefore we cannot account for attrition bias in our analyses. More variables could have been covered to account for social inequality in retirement transitions and to give a more nuanced picture of who the individuals are that continue in the workforce compared to the ones that retire. Unfortunately, this was not possible due to the pre-defined cohorts from the survey data. Further, respondents to the survey included more persons with a higher education and more persons in the workforce compared to the non-respondents [47]. On the same matter, the pre-defined cohorts consist of more males than females which is not a perfect representation of the Danish population. These aspects may lead to our results being skewed towards a socially selected group but we do not unfortunately have data for non-respondents in the scope of this project. However, we still believe that our results provide an important picture of who continues in the workforce past retirement age in Denmark. In the future, these aspects should be considered. Given the association between psychosocial and physical working conditions and retirement choices, which has already been documented in other Nordic countries [52, 53], more research is needed to uncover this relationship in the Danish context. Lastly, investigating the matter of causality in terms of the relationship between health and retirement by employing more robust analysis methods together with longitudinal data is a future priority.

## Conclusions

Careful consideration of individual differences should be applied when designing retirement policies. We find that individuals who continue in the workforce after retirement age have greater wealth, are in better health and are males compared to the retirees. The probability that an individual continues in the workforce past retirement age decreases with age, gender (female), years spent in the work force, alcohol consumption and physical activity. If the goal is to extend the working lives of older workers while maintaining flexibility and consideration for individual differences, abilities and capabilities, these results together with previous research in the field should be used as a guideline for designing retirement policies to encourage additional groups to work beyond retirement age.

### Electronic supplementary material

Below is the link to the electronic supplementary material.

Supplementary Material 1

## Data Availability

Data used in this study is not publicly available. In accordance with the Act on Processing of Personal Data (Act No. 429 of 31 May 2000) of the Danish Data Protection Agency, data cannot be made publicly available due to considerations for privacy and anonymity of the participants. However, the CAMB steering group welcomes collaboration and the interest of national and international colleagues. External researchers can get access to data by a collaboration agreement with the CAMB steering committee. For more information on how to apply, please contact the principal investigator Rikke Lund [camb@sund.ku.dk]. The CAMB home page [www.camb.ku.dk/Collaboration/] provides further information.

## Footnotes

1 Sources: https://ec.europa.eu/info/sites/default/files/economy-finance/final_country_fiche_dk_1.pdf, https://ec.europa.eu/social/main.jsp?catId=1107&intPageId=4494&langId=en, https://lifeindenmark.borger.dk/pension/state-pension, https://lifeindenmark.borger.dk/pension/early-retirement-pension,

2 The study population is based on self-reported information on retirement decisions from the survey data. No age cut-off was used when selecting individuals working after retirement or in full retirement due to reform induced differences in retirement age in Denmark (see Institutional Background).

## Abbreviations

BMI: Body Mass Index
CAMB: Copenhagen Aging and Midlife Biobank
CPR: Civil Registration Number
DALWUH: The Danish Longitudinal Study on Work, Unemployment, and Health cohort
VERP: Voluntary early retirement pension
