# Supplementary material for: Who continues to work after retirement age?
Source: BMC Public Health. 2024 Mar 4;24:692. doi: 10.1186/s12889-024-18161-1 (PMC10913677; doi:10.1186/s12889-024-18161-1)
Supplement: Supplementary file 1 — Supplementary Material 1 [file 12889_2024_18161_MOESM1_ESM.docx]

**Table A1. Correlation matrix over all variables included in the dataset**

|  | N=5012 |  |  |  |  |  |  |  |  |  |  |  |  |  |  |  |  |  |
| --- | --- | --- | --- | --- | --- | --- | --- | --- | --- | --- | --- | --- | --- | --- | --- | --- | --- | --- |
| **Variables** | Voluntary  work | Years  worked | Alcohol  consumption | Age | Gender | Children | Subordinates | Falls | Health | Physical  activity | Partner | Living  situation | Smoking | Education | Physical  Working  capability | Household  income | BMI | Partner’s  labormarket  status |
|  |  |  |  |  |  |  |  |  |  |  |  |  |  |  |  |  |  |  |
| Voluntary work | 1 |  |  |  |  |  |  |  |  |  |  |  |  |  |  |  |  |  |
| Years worked | 0.0015 | 1 |  |  |  |  |  |  |  |  |  |  |  |  |  |  |  |  |
| Alcohol | 0.0283 | -0.0058 | 1 |  |  |  |  |  |  |  |  |  |  |  |  |  |  |  |
| Age | 0.0387 | 0.3185 | -0.0158 | 1 |  |  |  |  |  |  |  |  |  |  |  |  |  |  |
| Gender | -0.0515 | 0.1206 | -0.1920 | 0.2052 | 1 |  |  |  |  |  |  |  |  |  |  |  |  |  |
| Children | -0.0192 | 0.0307 | 0.0179 | -0.0526 | -0.0501 | 1 |  |  |  |  |  |  |  |  |  |  |  |  |
| Subordinates | -0.0846 | -0.0190 | -0.0291 | -0.0311 | 0.0956 | 0.0376 | 1 |  |  |  |  |  |  |  |  |  |  |  |
| Falls | -0.0123 | -0.0143 | 0.0292 | -0.0457 | -0.0664 | 0.0002 | 0.0057 | 1 |  |  |  |  |  |  |  |  |  |  |
| Health | -0.0496 | 0.0566 | -0.0198 | 0.0509 | 0.0234 | 0.0606 | 0.0441 | -0.1805 | 1 |  |  |  |  |  |  |  |  |  |
| Phy.activity | 0.0333 | -0.0128 | -0.0034 | 0.0541 | 0.0550 | -0.0436 | -0.0136 | 0.0461 | -0.2233 | 1 |  |  |  |  |  |  |  |  |
| Partner | -0.0000 | -0.0332 | 0.0219 | -0.0501 | -0.0851 | -0.1693 | -0.0739 | 0.0536 | -0.1519 | 0.0987 | 1 |  |  |  |  |  |  |  |
| Living sit. | 0.0079 | -0.0250 | -0.0001 | -0.0353 | -0.0678 | -0.1734 | -0.0700 | 0.0550 | -0.1367 | 0.0949 | 0.8554 | 1 |  |  |  |  |  |  |
| Smoking | 0.0459 | 0.0036 | -0.0839 | 0.0526 | 0.0389 | -0.0509 | -0.0173 | 0.0182 | -0.0936 | 0.0802 | 0.1323 | 0.1288 | 1 |  |  |  |  |  |
| Education | 0.0177 | -0.1296 | 0.0360 | -0.0544 | -0.0154 | -0.0159 | -0.0761 | 0.0211 | -0.1318 | 0.0809 | 0.0869 | 0.0595 | 0.0743 | 1 |  |  |  |  |
| Phy.work.c. | -0.0374 | 0.0253 | -0.0185 | -0.0284 | 0.0270 | 0.0394 | -0.0005 | -0.1633 | 0.3673 | -0.1651 | -0.0864 | -0.0806 | -0.0680 | -0.0803 | 1 |  |  |  |
| H.income | -0.0148 | -0.1743 | 0.0408 | -0.2668 | -0.1063 | -0.1040 | -0.1011 | 0.0648 | -0.2245 | 0.0824 | 0.2621 | 0.2629 | 0.1191 | 0.3252 | -0.1492 | 1 |  |  |
| BMI | 0.0039 | 0.0177 | 0.0185 | -0.0153 | -0.0894 | 0.0026 | 0.0202 | -0.0271 | 0.2038 | -0.1567 | -0.0442 | -0.0375 | 0.0577 | -0.1706 | 0.0813 | -0.1194 | 1 |  |
| Partner.l.stat. | 0.0196 | 0.1665 | -0.0058 | 0.3416 | 0.1318 | 0.0595 | -0.0135 | -0.0073 | 0.0705 | 0.0290 | -0.2619 | -0.1938 | -0.0031 | -0.0577 | 0.0155 | -0.3845 | 0.0071 | 1 |
